# Educational inequalities in mortality and associated risk factors: German- versus French-speaking Switzerland

**DOI:** 10.1186/1471-2458-10-567

**Published:** 2010-09-22

**Authors:** David Faeh, Matthias Bopp

**Affiliations:** 1 Institute of Social and Preventive Medicine (ISPM), University of Zurich, Hirschengraben 84, 8001 Zurich, Switzerland

## Abstract

**Background:**

Between the French- and German-speaking areas of Switzerland, there are distinct differences in mortality, similar to those between Germany and France. Assessing corresponding inequalities may elucidate variations in mortality and risk factors, thereby uncovering public health potential. Our aim was to analyze educational inequalities in all-cause and cause-specific mortality in the two Swiss regions and to compare this with inequalities in behavioural risk factors and self-rated health.

**Methods:**

The Swiss National Cohort, a longitudinal census-based record linkage study, provided mortality and survival time data (3.5 million individuals, 40-79 years, 261,314 deaths, 1990-2000). The Swiss Health Survey 1992/93 provided cross-sectional data on risk factors. Inequalities were calculated as percentage of change in mortality rate (survival time, hazard ratio) or risk factor prevalence (odds ratio) per year of additional education using multivariable Cox and logistic regression.

**Results:**

Significant inequalities in mortality were found for all causes of death in men and for most causes in women. Inequalities were largest in men for causes related to smoking and alcohol use and in women for circulatory diseases. Gradients in all-cause mortality were more pronounced in younger and middle-aged men, especially in German-speaking Switzerland. Mortality inequalities tended to be larger in German-speaking Switzerland whereas inequalities in associated risk factors were generally more pronounced in French-speaking Switzerland.

**Conclusions:**

With respect to inequalities in mortality and associated risk factors, we found characteristic differences between German- and French-speaking Switzerland, some of which followed gradients described in Europe. These differences only partially reflected inequalities in associated risk factors.

## Background

Social inequalities in all-cause and cause-specific mortality have been reported for many European countries, including Switzerland [1,2]. However, in general there is a serious lack of comparable data [3]. Comparisons between countries are also hampered by the prevailing use of aggregated instead of individual data. Even when adequate data are available, it can still be difficult to assess inequalities because of nationally different definitions of socio-economic status (SES) and substantial variation regarding assignment of causes of death or assessment of risk factors (including self-rated health) between countries [4-6]. The coarse definition and classification of SES makes it often difficult to compare inequality in mortality from specific causes. This lack of comparable data precludes exploring possible explanations for inequalities in mortality.

Regional comparisons may identify potential for reduction of inequalities. Switzerland offers a unique setting because it combines cultural diversity within common health care and statistical systems. A recent comparison showed characteristic variations in cause-specific mortality and risk factors between the German- and French-speaking areas of Switzerland reflecting broader European patterns [7]. Such variations indicate unexploited potential for reduction of health inequalities. Regional differences in socioeconomic characteristics are minor. Compared to German-speaking areas, in French-speaking Switzerland there is a slightly lower proportion of persons with intermediate education and a higher proportion with high and low education (see Additional file 1, Table S2). Economic and wealth parameters are comparable, except of a slightly higher unemployment rate in French-speaking Switzerland.

We sought to elucidate the contribution of SES inequalities in mortality and risk factors to regional differences in mortality and to look for parallels in European populations. To date, no such comparison of inequality between the two major linguistic regions of Switzerland is available. The purpose of this study was to analyse variations between the French- and German-speaking areas of Switzerland in educational inequalities in 1) cause-specific mortality and to compare them with 2) inequalities in associated risk factors. We used two different data sets: 1) census linked mortality data and 2) health survey data.

## Methods

### Assessment of Mortality: Swiss National Cohort

The Swiss National Cohort is a national longitudinal research platform based on anonymous record linkage of data collected by the Swiss Federal Statistical Office. The core cohort consists of the 6.874 million residents who participated in the 1990 census and were evaluated for a linkage with the 2000 census, a mortality or an emigration record; for 93.1% of this population a satisfactory link could be established (for more details see [8]). The 1990 and 2000 censuses in Switzerland were carried out with self-administered questionnaires. Non-participation is considered to be very low (coverage for 2000 census: 98.6%) [9]. Of all registered deaths between the 1990 and the 2000 censuses, 95.3% could be successfully linked to the Swiss National Cohort [10]. For this study individuals were followed up in the period between Dec 4, 1990, and Dec 5, 2000 (census dates). Because of low mortality at young age and strong survival selection and difficulties in unicausal assignment of causes of death at oldest age, we limited the analyses to those aged between 40 and 79. We did not impose a limit at 65 because some risk factors have a very long latency and limitation would have hampered comparison of inequalities in mortality and risk factors. Deaths and person years were accumulated only for that age group. Thus, the youngest observed subject had just passed his 30th birthday on Dec 4, 1990, and contributed only one day of observation on Dec 4, 2000. Individuals aged 80 and older at the 1990 census were excluded and those reaching their 80th birthday between the census dates were censored. Overall, we included all Swiss and foreign nationals satisfactorily linked to a mortality or 2000 census record and living in the German-speaking (GS, n = 2,629,271) or French-speaking areas of Switzerland (FS, n = 820,849), respectively. Because cell sizes were too small, we had to exclude the Italian-speaking areas of the country (less than 5% of total population).

### Assessment of SES

For the determination of SES, we used two approaches. For regression analysis, we used level and estimated years of education (between 8 and 19 years respectively, for details see Additional file 1, Table S1). For descriptive mortality and prevalence (%) rates, we reclassified the denotations used in the census and the survey into three educational categories: 1) less than secondary: compulsory schooling or less (International Standard Classification of Education, ISCED 1-2); 2) secondary: vocational training or high school (ISCED 3-4); 3) tertiary: technical colleges and upper vocational education and university education (ISCED 5-6) [11]. Mean years of education by educational category and proportion by year of education are shown in the Additional file 1, Table S1.

### Assessment of risk factors

Data on risk factors stem from the first Swiss Health Survey 1992/1993. The health survey is a cross-sectional, nationwide, population-based telephone survey conducted every 5 years by the Swiss Federal Statistical Office to monitor public health trends. The survey was completed by 15,288 participants (15 years and older, 71% participation rate, 52% women, for more details see [12]).

In line with the mortality analysis, we limited the age range to 40-79 years for a total of 7,378 persons (for more details see Additional file 1, Table S2). For simplicity, we defined only risk (and not protective) factors with alcohol as having positive and negative effects on health. Risk factors were defined as follows: "Current smoking" when smoking ≥ 1 cigarette/day; "daily alcohol consumption" when drinking alcohol at least once per day; "infrequent fruit consumption" when not eating fruits daily; "physical inactivity" when not sweating at least once per week by performing physical activity in leisure time. Obesity was defined as Body Mass Index (= weight/(height)^2^) ≥ 30 kg/m^2^. Although self-rated health may be regarded as a risk marker and not as a risk factor per se, we treat it like a risk factor in our study. Possible answers for rating one's own health were "very good", "good", "fair", "poor", "very poor". We subsumed the latter three into a single category "less-than-good health".

### Statistical analysis

Age-standardized mortality rates and prevalence rates were calculated as described [7,13]. We performed analyses on the main groups of causes of death (diseases of the circulatory system, cancers, other diseases, and injuries) and on causes related to smoking and alcohol consumption. Types of cancers included upper aerodigestive tract (UADT: oropharynx, larynx, oesophagus), stomach, intestine, liver, lung, prostate and female breast. Table S3 (Additional file 1) shows the ICD (International Classification of Diseases) 8/9 and ICD 10 codes of selected causes of death. In Switzerland ICD-8 was used until 1994 followed by ICD-10 thereafter.

Since categorization of education into three levels loses valuable information, we used years of education instead. We calculated the relative change of hazard ratios (mortality rates) and odds ratios (risk factors prevalence), with every year of education in addition to lowest education (8 years). This was done using individual data and Cox regression (for modelling survival in cohort data) and logistic regression (for modelling the prevalence of risk factors in the health survey data) with years of education as independent variable. For the analysis of the health survey data we added age (linear and quadratic; the quadratic term was introduced to allow for a progressive increase of mortality with age) to the model and weighted proportions to the Swiss population. The STATA command "stcox" automatically adjusts for age (linear and exponential). In order to quantify differences (with p-values) in educational gradients between German and French-speaking Switzerland, we included in the model, in addition to the region variable, an interaction term (region times years of education).

As shown in Additional file 1, Table S1, with respect to demographic characteristics, there are only small differences between the cohort population and the health survey sample (except for those with tertiary education). As sensitivity analysis, we calculated the Relative Index of Inequality (RII) [1,14]. The RII provided a very similar pattern of inequality but larger confidence intervals. Therefore, we preferred the year-of-education-approach. The use of RII is necessary when data are aggregated or when parameters of inequality differ as is often the case between countries. With individual data and a uniform definition of SES, we could better exploit data with the year-of-education approach and avoid the loss of information. With both the cohort and health survey data, we also performed analysis excluding foreign nationals which did not change estimates significantly (not shown). Analyses were performed with STATA SE (Version 9, StataCorp, College Station, TX).

## Results

Overall 3,450,120 persons accumulating 26,734,458 person-years were included: 1,278,052 men and 1,351,219 women from GS; and 392,451 men and 428,398 women from FS. Details are shown in the Additional file 1, Table S2.

### Inequality in mortality

**Figure 1:**
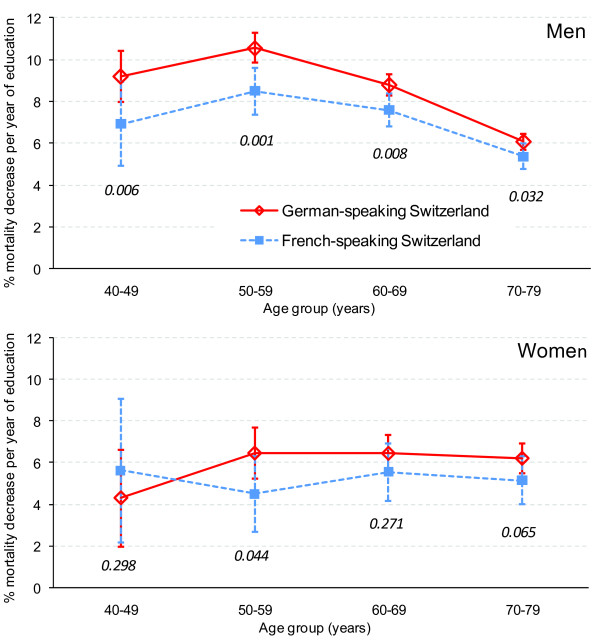
**Relative decrease of all-cause mortality per year of additional education**. Estimates are % with 95% confidence interval, by sex and region, 1990-2000. Figures in italic are p-values for the statistical significance of the difference between Swiss regions in decrease of mortality per additional year of education. Data source: Swiss Federal Statistical Office/Swiss National Cohort.

As shown in figure 1, all-cause mortality per additional year of education decreased in the youngest age group by about 7% (FS) and 9% (GS) in men and by about 6% (FS) and 4% (GS) in women. In GS men aged 50-59 years, those with the lowest education (11 years of maximum education difference with 10.51% increase per year of education: 1.1051 to the power of 11 = 3.0) had a three times higher all-cause mortality risk compared to those with the highest education. In men but not in women, inequalities appeared to decrease with age. In men, inequalities were significantly larger in GS than in FS in all age groups. This was also true in women except for the youngest age group.

Table 1 and 2 show counts and mortality rates (per 100,000 person-years) for selected causes of death by region and sex, overall and by educational level. The tables also give the percentage change of mortality rate for each additional year of education. Almost all of the educational gradients were mathematically monotonical. In men, there was a significant educational gradient for all causes of death in both regions except for prostate cancer in FS. The largest inequalities were found for UADT, stomach and lung cancer, COPD and liver cirrhosis. In women significant inequality gradients (confidence intervals excluded zero) were less frequent and generally restricted to circulatory disease, stomach cancer and alcohol-(liver cirrhosis) or smoking-related (COPD, lung cancer) causes of death. In GS, ill-defined causes and suicide showed a significant inverse gradient.

**Table 1:** Inequality in mortality in Switzerland by language region and cause of death in men

	German Switzerland (GS)							French Switzerland (FS)								
				
		Rate overall and by educational level				*Rate decrease per additional year of education*			Rate overall and by educational level				*Rate decrease per additional year of education*		GS vs. FS*	Significance of region*
								
	deaths	all	low	middle	high	*%*	*95% CI*	deaths	all	low	middle	high	*%*	*95% CI*	*p-value*	z-value
All causes	122182	1186	1515	1156	854	*7.74*	*(7.49;7.99)*	38721	1220	1478	1176	882	*6.45*	*(6.07;6.84)*	*< 0.001*	5.5
																
Circulatory system	44555	426	529	418	310	*7.29*	*(6.88;7.70)*	12141	376	451	363	272	*6.33*	*(5.64;7.02)*	*0.019*	2.36
Coronary heart disease	24732	238	283	239	177	*6.38*	*(5.84;6.92)*	5597	175	203	174	129	*5.31*	*(4.33;6.30)*	*0.062*	1.86
Other heart diseases	8297	80	108	76	52	*10.14*	*(9.14;11.15)*	1291	106	135	96	75	*7.97*	*(6.63;9.33)*	*0.012*	2.53
Stroke	6313	59	77	55	44	*7.93*	*(6.83;9.03)*	1735	53	62	52	37	*6.44*	*(4.62;8.29)*	*0.175*	1.36
																
Cancer	42872	420	513	419	315	*6.39*	*(5.98;6.80)*	14695	467	556	456	347	*5.96*	*(5.34;6.58)*	*0.250*	1.15
UADT cancer	3272	33	51	32	18	*15.20*	*(13.46;16.97)*	1604	53	70	53	28	*12.50*	*(10.34;14.69)*	*0.057*	1.9
Stomach cancer	2052	20	29	19	12	*12.84*	*(10.73;14.99)*	544	17	23	15	12	*9.18*	*(5.75;12.72)*	*0.081*	1.75
Intestinal cancer	4468	44	45	44	39	*1.89*	*(0.75;3.04)*	1467	46	50	48	39	*2.39*	*(0.63;4.19)*	*0.638*	-0.47
Liver cancer	1494	15	17	15	11	*6.18*	*(4.02;8.38)*	790	25	28	26	20	*4.27*	*(1.75;6.85)*	*0.265*	1.12
Lung cancer	11841	118	165	116	65	*12.97*	*(12.09;13.87)*	4163	134	179	123	82	*11.07*	*(9.76;12.40)*	*0.019*	2.34
Breast cancer	4852	45	47	45	43	*1.22*	*(0.14;2.30)*	1445	43	45	43	39	*1.66*	*(-0.09;3.45)*	*0.672*	-0.42
																
Other diseases	15497	149	212	139	97	*11.34*	*(10.76;11.92)*	4947	153	193	143	105	*7.63*	*(6.81;8.45)*	*< 0.001*	7.23
COPD	5418	51	80	45	24	*18.18*	*(16.73;19.64)*	1177	36	51	30	21	*13.89*	*(11.23;16.62)*	*0.007*	2.72
Liver cirrhosis	2490	25	37	25	15	*12.99*	*(11.08;14.94)*	1128	37	50	38	19	*12.20*	*(9.67;14.80)*	*0.630*	0.48
Ill-defined	2670	27	32	26	25	*1.72*	*(0.26;3.21)*	1832	58	64	59	50	*2.86*	*(1.26;4.48)*	*0.305*	-1.03
																
Injuries	8680	87	112	85	67	*6.44*	*(5.55;7.35)*	2801	91	113	87	68	*6.43*	*(5.03;7.85)*	*0.986*	0.02
Suicide	4121	42	46	42	36	*2.57*	*(1.37;3.78)*	1280	42	50	42	34	*4.42*	*(2.45;6.42)*	*0.116*	-1.57
Transport accident	1452	15	21	13	11	*11.46*	*(9.05;13.93)*	488	16	19	16	12	*4.94*	*(1.73;8.24)*	*0.002*	3.12
																
Smoking-related	20595	202	296	194	108	*14.72*	*(14.02;15.42)*	6969	223	301	206	131	*11.55*	*(10.52;12.58)*	*< 0.001*	4.98
Alcohol-related	8378	85	123	83	48	*12.96*	*(11.91;14.02)*	3867	127	163	128	73	*10.44*	*(9.11;11.78)*	*0.004*	2.91

*Statistical significance of the difference between Swiss regions in decrease of mortality per additional year of education

1990-2000, 40-79 years

UADT: Upper aerodigestive tract; COPD: Chronic obstructive pulmonary disease

ISCED: International Standard Classification of Education

Educational level: low = no secondary or tertiary education (ISCED 1-2), middle = secondary education (ISCED 3-4); high = tertiary education (ISCED 5-6)

Rates were standardized to the WHO standard population "Europe" [13]

Data source: Swiss Federal Statistical Office/Swiss National Cohort

**Table 2:** Inequality in mortality in Switzerland by language region and cause of death in women

	German Switzerland (GS)							French Switzerland (FS)								
				
		Rate overall and by educational level				*Rate decrease per additional year of education*			Rate overall and by educational level				*Rate decrease per additional year of education*		GS vs. FS*	Significance of region*
								
	deaths	all	low	middle	high	*%*	*95% CI*	deaths	all	low	middle	high	*%*	*95% CI*	*p-value*	z-value
All causes	77227	600	668	533	481	*6.17*	*(5.77;6.58)*	23184	572	629	502	462	*5.07*	*(4.42;5.72)*	*0.004*	2.84
																
Circulatory system	24935	177	209	141	110	*11.42*	*(10.61;12.23)*	6780	153	180	115	97	*11.20*	*(9.75;12.68)*	*0.801*	0.25
Coronary heart disease	10743	76	90	59	46	*12.13*	*(10.88;13.39)*	2368	53	65	37	29	*14.23*	*(11.58;16.95)*	*0.160*	-1.4
Other heart diseases	5223	37	44	31	24	*9.76*	*(8.06;11.48)*	2201	50	57	40	34	*8.99*	*(6.59;11.45)*	*0.612*	0.51
Stroke	2627	38	44	31	24	*10.22*	*(8.53;11.94)*	681	31	36	24	21	*9.18*	*(6.14;12.31)*	*0.562*	0.58
																
Cancer	30890	256	269	244	233	*2.46*	*(1.87;3.05)*	9464	248	259	240	225	*1.55*	*(0.65;2.46)*	*0.098*	1.66
UADT cancer	717	6	7	6	4	*4.19*	*(0.26;8.27)*	324	9	9	9	7	*3.40*	*(-1.56;8.60)*	*0.062*	1.87
Stomach cancer	1038	8	10	6	6	*13.61*	*(9.65;17.71)*	319	8	9	7	6	*6.87*	*(1.25;12.81)*	*0.547*	-0.6
Intestinal cancer	3165	25	25	25	24	*0.42*	*(-1.36;2.23)*	932	23	23	23	20	*0.27*	*(-2.53;3.15)*	*0.932*	0.09
Liver cancer	515	4	4	4	3	*3.75*	*(-0.91;8.63)*	195	5	6	4	4	*6.82*	*(-0.36;14.51)*	*0.490*	-0.69
Lung cancer	3189	28	30	27	23	*2.46*	*(0.64;4.31)*	1285	34	38	33	29	*4.06*	*(1.50;6.69)*	*0.317*	-1
Breast cancer	7146	63	61	63	66	*-1.01*	*(-2.14;0.13)*	2099	58	59	58	55	*0.41*	*(-1.38;2.23)*	*0.186*	-1.32
																
Other diseases	12898	96	113	80	70	*7.86*	*(6.98;8.76)*	3698	86	100	69	58	*7.64*	*(6.23;9.07)*	*0.791*	0.26
COPD	2047	15	18	13	9	*8.52*	*(5.89;11.22)*	505	12	13	10	9	*7.58*	*(2.84;12.54)*	*0.738*	0.33
Liver cirrhosis	1209	11	13	10	10	*5.40*	*(2.36;8.53)*	418	12	14	10	9	*9.62*	*(4.72;14.76)*	*0.153*	-1.43
Ill-defined	1331	11	11	11	17	*-4.97*	*(-7.36;-2.53)*	1014	26	27	24	26	*2.01*	*(-0.76;4.86)*	*< 0.001*	-3.75
																
Injuries	3917	34	35	33	33	*0.91*	*(-0.66;2.50)*	1305	35	36	34	39	*0.40*	*(-1.83;2.69)*	*0.718*	0.36
Suicide	1751	16	16	17	18	*-2.22*	*(-4.36;-0.03)*	574	17	17	17	19	*-1.17*	*(-4.28;2.05)*	*0.587*	-0.54
Transport accident	509	4	5	4	3	*2.61*	*(-1.82;7.25)*	205	5	5	6	7	*-2.45*	*(-7.57;2.95)*	*0.149*	1.44
																
Smoking-related	5980	49	54	46	36	*4.78*	*(3.37;6.20)*	2128	55	61	52	45	*4.25*	*(2.21;6.33)*	*0.678*	0.42
Alcohol-related	2861	25	28	24	20	*4.59*	*(2.61;6.61)*	1050	29	33	26	22	*7.44*	*(4.41;10.57)*	*0.123*	-1.54

*Statistical significance of the difference between Swiss regions in decrease of mortality per additional year of education

1990-2000, 40-79 years

ISCED: International Standard Classification of Education

UADT: Upper aerodigestive tract; COPD: Chronic obstructive pulmonary disease

Educational level: low = no secondary or tertiary education (ISCED 1-2), middle = secondary education (ISCED 3-4); high = tertiary education (ISCED 5-6)

Rates were standardized to the WHO standard population "Europe" [13]

Data source: Swiss Federal Statistical Office/Swiss National Cohort

In both regions, inequalities were larger in men than in women for mortality from all-causes, cancer (particularly UADT, stomach in FS and lung), COPD, liver cirrhosis, suicide and transport accidents, while they were larger in women than men for coronary heart disease (CHD) and stroke mortality.

Relative differences between FS and GS were more pronounced in men than in women. However, only few differences were significant (p < 0.05, in men: all causes, all circulatory, other heart diseases, lung cancer, COPD, transport accidents, alcohol and smoking-related causes; in women: all causes and ill-defined causes) or tended to be significant (p < 0.1, in men: coronary heart disease, UADT and stomach cancer; in women: all cancer and UADT cancer).

In men, the gradients were (or tended to be) steeper in GS than in FS for all 21 entities except for 5: suicide, intestinal and prostate cancer, ill-defined causes and all injuries. As in men, also in women, inequality in all-cause mortality and transport accidents was significantly larger in GS. However, inequality tended to be larger in FS than in GS for CHD, liver, lung and breast cancer, liver cirrhosis, suicide, ill-defined and alcohol-related causes.

### Inequality in risk factors

Table 3 shows the number of participants with risk factors, prevalence of risk factors overall and by educational level and relative change per year of education. Except for obesity in women, the prevalence of risk factors tended to be higher in FS than in GS, with the largest relative difference in daily fruit consumption. In men, inequalities in all risk factors were larger in FS, except for less-than-good health. In women, inequalities were larger in FS than in GS only for current smoking and infrequent fruit consumption. Inequalities were reversed ("negative") for alcohol consumption in women (i.e., more frequent consumption by those with higher education). The largest inequalities were found for obesity, particularly in women.

**Table 3:** Inequality in risk factors prevalence in Switzerland by language region and sex

	German-speaking Switzerland							French-speaking Switzerland							
			
		Prevalence (%) overall and by educational level				*Prevalence decrease per additional year of education*			Prevalence (%) overall and by educational level				*Prevalence decrease per additional year of education*		GS vs. FS*
							
	N	all	low	middle	high	*%*	*95% CI*	N	all	low	middle	high	*%*	*95% CI*	*p-value*
Men (N = 3,241)															
Current smoking	579	23	21	26	18	*6.46*	*(6.27;6.66)*	260	28	32	30	24	*6.99*	*(6.70;7.29)*	*0.004*
Daily alcohol consumption	815	35	41	35	31	*4.08*	*(3.91;4.24)*	465	52	56	54	45	*4.80*	*(4.55;5.05)*	*< 0.001*
Infrequent fruit consumption	719	26	31	30	29	*2.39*	*(2.22;2.55)*	338	30	33	38	33	*4.66*	*(4.40;4.93)*	*< 0.001*
Physical inactivity	812	33	42	35	27	*7.12*	*(6.95;7.30)*	336	36	45	37	29	*8.52*	*(8.24;8.80)*	*< 0.001*
Obesity	198	9	15	8	8	*12.80*	*(12.5;13.1)*	82	8	13	8	5	*18.12*	*(17.6;18.7)*	*< 0.001*
															
Less-than-good health	394	16	21	18	11	*7.38*	*(7.15;7.61)*	171	19	30	17	14	*7.32*	*(6.97;7.67)*	*0.769*
															
Women (N = 4,137)															
Current smoking	566	19	19	18	22	*4.30*	*(4.05;4.54)*	285	22	23	24	17	*8.32*	*(7.98;8.67)*	*< 0.001*
Daily alcohol consumption	311	10	9	10	14	*-7.76*	*(-8.00;-7.50)*	320	28	23	33	23	*-3.30*	*(-3.67;-3.03)*	*< 0.001*
Infrequent fruit consumption	471	10	15	14	12	*5.38*	*(5.10;5.70)*	308	20	29	22	23	*6.09*	*(5.77;6.41)*	*0.001*
Physical inactivity	1209	39	47	35	36	*9.17*	*(8.96;9.38)*	555	47	53	46	35	*7.52*	*(7.24;7.80)*	*< 0.001*
Obesity	214	7	11	6	3	*27.01*	*(26.5;27.5)*	80	6	10	5	3	*26.99*	*(26.3;27.7)*	*0.960*
															
Less-than-good health	588	19	25	16	12	*12.50*	*(12.2;12.8)*	319	27	35	22	22	*8.70*	*(8.36;9.05)*	*< 0.001*

*Statistical significance of the difference between Swiss regions in decrease of mortality per additional year of education

1992/93, 40-79 years

N: Number of persons reporting respective risk factors

Educational level: low = no secondary or tertiary education (ISCED 1-2), middle = secondary education (ISCED 3-4); high = tertiary education (ISCED 5-6)

Data source: Swiss Federal Statistical Office, Swiss Health Survey 1992/93

## Discussion

We examined educational inequalities in mortality, related risk factors (including self-rated health) in the French- and German-speaking areas of Switzerland. In both regions, the largest inequalities were found in men in mortality from causes related to smoking and alcohol consumption, and in women from circulatory diseases and stomach cancer. Mortality inequalities tended to be larger in German than in French-speaking Switzerland (particularly in men). Regional inequality differences in the corresponding risk factors generally failed to explain variations in mortality inequalities. In both regions, inequality in mortality was larger than could be expected from inequalities in associated risk factors. We found large inequalities in mortality from alcohol-related causes, such as liver cirrhosis, UADT and liver cancer. Particularly in women, these inequalities were at odds with the low or reversed inequality in alcohol consumption. In France and Spain, inequality in alcohol-related cancer mortality was similar and could also not be explained by inequalities in alcohol consumption [15-17]. Possibly, telephone interviews may not be the best method to assess the full range of alcohol consumption. Moreover, persons with hazardous alcohol consumption may more often refuse to participate in health surveys than moderate drinkers or abstainers. In our study, we used inequalities in daily alcohol consumption (yes or no). However, for a part of the participants (72%), the amount of ethanol consumed daily was available. A mere 3.4% reported daily intake that exceeded 60 g of ethanol, and 16% were abstainers. The inequality pattern resulting from alcohol consumers in the upper quintile (men > 71 g, women > 27 g) was similar to that of daily alcohol consumption (table 3) in men but not in women. In GS women, inequalities were not reversed (negative) as was the case for daily alcohol consumption, but positive (5% decrease of those in the upper quintile per additional year of education). This could explain the larger inequality in UADT cancer in GS women.

In our study, the large inequalities in stomach cancer mortality in both regions could not be explained by similar inequalities in infrequent consumption of fruits, particularly in men. In contrast, in Germany, inequality in stomach cancer mortality in the late 1980 s could be explained by similarly large inequalities in fruit consumption [15,18]. We could not consider risk factors which are more specific for stomach cancer, such as Helicobacter pylori [19]. This bacteria is more likely to affect persons with low than with high SES [20]. Because of the increasing availability and affordability of fruits and vegetable, improved sanitary conditions and better diagnosis and treatment of Helicobacter pylori, inequalities in stomach cancer could further decrease [21].

In contrast to smoking and obesity, alcohol consumption can also be regarded as a protective factor against cardiovascular disease, particularly when consumed in a context of a healthy lifestyle [22,23]. However, when comparing FS and GS, inequalities in these risk factors did not consistently fit to inequalities in CHD mortality.

In line with the broader European pattern, in both regions, the relatively small inequalities in infrequent fruit consumption and physical inactivity failed to entirely explain variations in inequalities in CHD which were comparably large, particularly in women [1,15,17,21]. This could be due to the "healthy participant effect" of health surveys, to risk factor assessment or be a consequence of inequalities in treatment and diagnosis: For "harder" cardiovascular risk factors, such as high blood pressure, much larger inequalities in women than in men were found in Spain. Also, after cardiac surgery, SES inequalities in survival remained significant after adjustment for smoking, obesity and diabetes [24]. Several authors explained the significantly larger inequality in circulatory disease mortality in women compared to men by the poorer diagnosis and treatment in women with cardiovascular disease [25-27].

In both regions and sexes, smoking inequalities were comparably small and failed to explain the large gradient in lung cancer mortality, particularly in men. Larger inequalities in smoking and in lung cancer mortality in men than in women are typical for southern European countries [1,21,28,29]. We found this pattern predominantly in women, where inequalities in lung cancer tended to be larger in FS than in GS. For several reasons, we assume that in men the relation between smoking inequality and inequality in lung cancer mortality is underestimated in our figures. First, smokers and persons belonging to lower SES more often refuse to participate in health surveys than non-smokers [30]. Participating smokers may therefore represent a strongly selected population which is possibly also more prone to report social desirable behaviours [30]. Second, other risk factors for lung cancer are more frequent in persons in lower SES (e.g. air pollution, occupational exposures) and thus contribute to inequality in mortality [29]. Third, diagnosis and treatment possibly are better in those with higher SES irrespective of risk factors [24,31]. Thus, there may be a "double social inequality" in persons with low SES, arising from a higher risk for lung cancer and, once affected, a higher risk of dying from the disease [18]. Similar patterns also apply for inequality in COPD mortality, which was also large in women. As in lung cancer, international differences in COPD mortality could not be explained by differences in smoking [32]. In addition to above-mentioned factors, the increased risks of respiratory disease in farmers could map to the educational gradients [33].

Educational inequality in suicide may correspond to the European average for both sexes [34]. In contrast, in men, inequalities in transport accident mortality were larger in Switzerland than in most other European countries [35]. The relatively large inequality in mortality from transport accidents in men in both GS and FS as well as the substantial difference between these regions are in line with the large inequality in mortality from other causes of death related to alcohol consumption.

Self-rated health is a good predictor of mortality [36,37]. The inequality pattern (women > men) in less-than-good self-rated health could also be found on a European scale [1]. However, differences between GS and FS were much smaller than those between Germany and France [1]. Larger inequalities in less-than-good self-rated health in GS women were in line with larger inequalities in all-cause mortality. In accordance with the larger inequality in GS women, in Europe, inequalities in self-rated health are largest in countries with more egalitarian health policies such as Norway or the Netherlands [38]. In contrast to studies comparing northern and southern European countries, we have no evidence of different perceptions of health between the language regions [11,39,40].

As shown in our previous publication, there were substantial variations in cause-specific mortality between Swiss regions and similar variations could be found on a broader European scale [7]. When a specific cause of death is more prevalent in one region than in another, one could expect unexploited potential for reduction of health inequalities. In our data, this has been confirmed by the fact that higher mortality from COPD in GS than in FS was accompanied by larger inequality in COPD mortality. Accordingly, higher mortality rates of alcohol-related causes (UADT and liver cancer and liver cirrhosis) found in FS would lead one to expect larger inequalities in that region, but this was not the case. This discordance may relate to the fact that predominantly hazardous but not moderate drinking is responsible for cancer and cirrhosis. Hazardous drinking is only poorly captured by a questionnaire used in a health survey and reporting probably differs substantially, depending on cultural and social norms [41]. In addition to alcohol consumption, self-rated health as well as reporting height and weight (influencing obesity prevalence) may vary strongly between cultures [39,42]. In our previous study, only part of the variation in mortality between GS and FS could be explained by differences in risk factors [7].

In addition to inequality in risk factors, inequality in mortality could also be biased e.g., when assignment of causes of death depends on SES or on cultural peculiarities [7]. Comparisons between risk factors and mortality inequality should be interpreted cautiously. Most risk factors impact on mortality with a latency of 20 years or more. Thus, simultaneous assessments of inequality in mortality and risk factors only roughly reflect real associations. Also, on a population base, only cross-sectional (1992/93) data on risk factors was available precluding interpretation of trends in risk factors and corresponding inequalities. However, comparisons with earlier (1977) and later (2002) assessments suggest that the prevalence of many major risk factors remained quite stable in the Swiss population [43,44]. Another limitation for comparison is coverage. The Swiss National Cohort covers the whole population and is based on mandatory data collection (census, vital statistics), whereas the health survey is a 3‰ sample with a participation rate of 71%. Persons with an unhealthy lifestyle or belonging to lower socioeconomic groups are likely to be underrepresented in health surveys [30]. In fact, there are more persons with tertiary education in the survey than in the cohort which may hamper gradient comparison. A potential source of bias is also the use of education for determining inequality. In the Swiss federal system, there are in fact not only one but 26 educational systems. However, these variations coincide only partially with language regions and are much smaller than between countries.

## Conclusions

Educational inequalities in mortality were substantial in Switzerland, particularly for causes with known risk factors (i.e., smoking, alcohol consumption, and obesity). In contrast, inequalities in most of these risk factors per se were smaller than expected. Since risk factors often occur together (cluster), their joint impact may better correspond to the inequality in mortality. In accordance with the broader European pattern, most mortality inequalities were larger in German- than in French-speaking areas. However, this was at odds with generally larger inequalities in risk factors in French-speaking Switzerland. This discrepancy may be due to weaknesses in risk factor assessment and assignment of cause of death.

## Competing interests

The authors declare that they have no competing interests.

## Authors' contributions

MB conceived the study and sketched a first draft. MB and DF prepared the data file of the Swiss National Cohort, DF prepared the data file of the Swiss Health Survey 1992/93. DF wrote up the manuscript. Literature review, data analysis and interpretation of the results were performed by both authors, as well as repeated editing of the manuscript. Both authors read and approved the final manuscript.

## Pre-publication history

The pre-publication history for this paper can be accessed here:

http://www.biomedcentral.com/1471-2458/10/567/prepub

## Supplementary Material

Additional file 1 **Appendix**. Table S1 - Comparison of study populations, by sex and language region. Table S2 - Individuals included at 1990 census by region and sex, overall and % by educational level. Table S3 - List of causes of death. Click here for file
