# Supplementary material for: Educational inequalities in mortality and associated risk factors: German- versus French-speaking Switzerland
Source: BMC Public Health. 2010 Sep 22;10:567. doi: 10.1186/1471-2458-10-567 (PMC2955004; doi:10.1186/1471-2458-10-567)
Supplement: Additional file 1 — Appendix. Table S1 - Comparison of study populations, by sex and language region. Table S2 - Individuals included at 1990 census by region and sex, overall and % by educational level. Table S3 - List of causes of death. [file 1471-2458-10-567-S1.DOC]

**Appendix**

**Table S1 - Comparison of study populations, by sex and language region**

|  |  |  |  |  |  |
| --- | --- | --- | --- | --- | --- |
|  | Men | |  | Women | |
|  | SNC | SHS |  | SNC | SHS |
| **German Switzerland** |  |  |  |  |  |
| % Sex | 47.7 | 44.1 |  | 52.3 | 55.9 |
| Mean age (years) | 55.5 | 55.9 |  | 57.0 | 57.1 |
| % Foreign nationals | 15.0 | 13.7 |  | 8.8 | 10.5 |
| Mean years of education by level |  |  |  |  |  |
| Low (no secondary education, ISCED 1-2) | 8.9 | 8.9 |  | 8.9 | 9.0 |
| Middle (secondary education, ISCED 3-4) | 12.0 | 12.1 |  | 12.1 | 12.1 |
| High (tertiary education, ISCED 5-6) | 17.0 | 15.9 |  | 17.1 | 15.6 |
| All | 12.4 | 12.9 |  | 11.1 | 11.5 |
| % by estimated number of years of education |  |  |  |  |  |
| 8: No secondary (ISCED 1-2), Incomplete compulsory school or no education | 2.9 | 0.7 |  | 4.1 | 1.0 |
| 9: No secondary (ISCED 1-2), Compulsory school | 21.8 | 13.1 |  | 43.4 | 30.5 |
| 12: Secondary (ISCED 3-4), Vocational education | 51.8 | 50.0 |  | 43.3 | 52.0 |
| 13: Secondary (ISCED 3-4), High School | 1.9 | 3.3 |  | 3.9 | 4.2 |
| 14: Tertiary (ISCED 5-6), Upper vocational education | 10.0 | 15.0 |  | 2.6 | 7.1 |
| 16: Tertiary (ISCED 5-6), technical college | 4.2 | 8.1 |  | 0.9 | 2.2 |
| 19: Tertiary (ISCED 5-6), Graduate school (university) | 7.5 | 9.8 |  | 1.8 | 3.0 |
| **French Switzerland** |  |  |  |  |  |
| % Sex | 47.0 | 43.4 |  | 53.0 | 56.6 |
| Mean age (years) | 55.4 | 55.1 |  | 56.7 | 56.8 |
| % Foreign nationals | 23.7 | 22.5 |  | 15.5 | 15.7 |
| Mean years of education by level |  |  |  |  |  |
| Low (no secondary education, ISCED 1-2) | 8.9 | 9.0 |  | 8.9 | 9.0 |
| Middle (secondary education, ISCED 3-4) | 12.1 | 12.1 |  | 12.2 | 12.1 |
| High (tertiary education, ISCED 5-6) | 17.4 | 16.5 |  | 17.5 | 16.3 |
| All | 12.3 | 12.8 |  | 11.1 | 11.5 |
| % by estimated number of years of education |  |  |  |  |  |
| 8: No secondary (ISCED 1-2), Incomplete compulsory school or no education | 2.9 | 0.4 |  | 3.7 | 1.3 |
| 9: No secondary (ISCED 1-2), Compulsory school | 30.7 | 19.0 |  | 49.6 | 38.4 |
| 12: Secondary (ISCED 3-4), Vocational education | 40.7 | 47.1 |  | 32.4 | 38.6 |
| 13: Secondary (ISCED 3-4), High School | 2.7 | 4.2 |  | 5.8 | 6.5 |
| 14: Tertiary (ISCED 5-6), Upper vocational education | 8.8 | 11.0 |  | 3.6 | 6.4 |
| 16: Tertiary (ISCED 5-6), technical college | 3.4 | 5.6 |  | 0.8 | 3.7 |
| 19: Tertiary (ISCED 5-6), Graduate school (university) | 10.7 | 12.6 |  | 4.1 | 5.2 |

ISCED: International Standard Classification of Education

Data source: Swiss Federal Statistical Office, Swiss National Cohort (SNC) and Swiss Health Survey 1992/93 (SHS)

**Table S2 - Individuals enumerated in the 1990 census by region and sex, overall and % by educational level**

|  |  |  |  |  |  |  |  |  |  |
| --- | --- | --- | --- | --- | --- | --- | --- | --- | --- |
|  | German Switzerland | | | |  | French Switzerland | | | |
|  |  | % by educational level | | |  |  | % by educational level | | |
| Age | N | low | middle | high |  | N | low | middle | high |
| Men |  |  |  |  |  |  |  |  |  |
| 30-34 | 191,181 | 21 | 55 | 24 |  | 58,188 | 28 | 48 | 24 |
| 35-39 | 176,758 | 20 | 54 | 26 |  | 52,482 | 26 | 47 | 27 |
| 40-44 | 177,898 | 19 | 55 | 26 |  | 55,003 | 25 | 48 | 27 |
| 45-49 | 162,244 | 18 | 56 | 26 |  | 50,959 | 25 | 47 | 28 |
| 50-54 | 128,552 | 22 | 55 | 23 |  | 39,658 | 30 | 45 | 25 |
| 55-59 | 117,417 | 23 | 56 | 21 |  | 36,405 | 33 | 45 | 22 |
| 60-64 | 104,078 | 26 | 54 | 20 |  | 32,188 | 38 | 42 | 20 |
| 65-69 | 92,402 | 34 | 48 | 18 |  | 29,539 | 45 | 37 | 18 |
| 70-74 | 70,759 | 36 | 48 | 16 |  | 21,452 | 47 | 36 | 17 |
| 75-79 | 56,763 | 38 | 49 | 14 |  | 16,577 | 49 | 36 | 15 |
| 30-79 | 1,278,052 | 25 | 54 | 21 |  | 392,451 | 35 | 43 | 23 |
| Women | |  |  |  |  |  |  |  |  |
| 30-34 | 182,740 | 27 | 63 | 10 |  | 56,849 | 33 | 53 | 15 |
| 35-39 | 170,495 | 30 | 60 | 10 |  | 53,473 | 35 | 51 | 14 |
| 40-44 | 170,721 | 33 | 59 | 8 |  | 56,963 | 38 | 49 | 13 |
| 45-49 | 157,089 | 35 | 57 | 8 |  | 51,921 | 40 | 48 | 12 |
| 50-54 | 132,408 | 42 | 52 | 6 |  | 40,910 | 48 | 42 | 10 |
| 55-59 | 126,249 | 49 | 47 | 4 |  | 39,580 | 56 | 37 | 7 |
| 60-64 | 120,822 | 54 | 42 | 4 |  | 37,664 | 62 | 32 | 6 |
| 65-69 | 114,176 | 59 | 38 | 4 |  | 36,514 | 66 | 28 | 6 |
| 70-74 | 91,375 | 62 | 35 | 3 |  | 28,415 | 69 | 27 | 4 |
| 75-79 | 85,144 | 67 | 31 | 2 |  | 26,109 | 72 | 25 | 4 |
| 30-79 | 1,351,219 | 49 | 46 | 5 |  | 428,398 | 55 | 37 | 8 |

ISCED: International Standard Classification of Education

Educational level: low = no secondary or tertiary education (ISCED 1,2), middle = secondary education (ISCED 3,4); high = tertiary education (ISCED 5,6)

Data source: Swiss Federal Statistical Office / Swiss National Cohort

**Table S3 - List of causes of death**

|  |  |  |
| --- | --- | --- |
| Cause of death | ICD 8 | ICD 10 |
| Circulatory system |  |  |
| Coronary heart disease | 410-414 | I20-I25 |
| Other heart diseases | 420-429 | I26-I52 |
| Stroke | 430-438 | I60-I69 |
| Cancer |  |  |
| UADT cancer | 140-150; 161 | C00-C15; C32 |
| Stomach cancer | 151 | C16 |
| Intestinal cancer | 152-154 | C17-C21 |
| Liver cancer | 155 | C22 |
| Lung cancer | 162 | C33; C34 |
| Prostate cancer | 185 | C61 |
| Breast cancer | 174 | C50 |
| Other disease |  |  |
| COPD | 490-493 | J40-J47 |
| Liver cirrhosis | 571 | K70; K74 |
| Ill-defined | 780-799 | R00-R99 |
| Injury and poisoning |  |  |
| Suicide | 950-959 | X60-X84 |
| Transport accident | 800-845 | V00-V99 |
| Smoking-related | 140-150; 160-162; 490-493 | C00-C15; C30-C34; C39; J40-J47 |
| Alcohol-related | 140-150; 155; 161; 291; 303; 571; 577; 860 | C00-C15; C22; C32; F10; I42.6; K70; K74; K85-K86; X45 |

ICD: International Classification of Diseases

UADT: Upper aerodigestive tract (oropharynx, larynx, oesophagus); COPD: Chronic obstructive pulmonary disease
